# Strategies to Improve the Clinical Outcomes for Direct-to-Consumer Pharmacogenomic Tests

**DOI:** 10.3390/genes12030361

**Published:** 2021-03-03

**Authors:** Alireza Tafazoli, Rama Krishna Guggilla, Zahra Kamel-Koleti, Wojciech Miltyk

**Affiliations:** 1 Department of Analysis and Bioanalysis of Medicines, Faculty of Pharmacy with the Division of Laboratory Medicine, Medical University of Bialystok, 15-089 Bialystok, Poland; wojciech.miltyk@umb.edu.pl; 2 Clinical Research Centre, Medical University of Bialystok, 15-276 Bialystok, Poland; 3 Department of Population Medicine and Civilization Diseases Prevention, Faculty of Medicine with the Division of Dentistry and Division of Medical Education in English, Medical University of Bialystok, 15-269 Bialystok, Poland; rama.guggilla@umb.edu.pl; 4 Department of Pathology and Medical Laboratory, Shohada Hospital, Mazandaran University of Medical Sciences, Behshahr 4851613185, Iran; 00zahra.kaamel00@gmail.com

**Keywords:** direct-to-consumer pharmacogenomic tests, clinical related outcome, personalized medicine

## Abstract

Direct-to-consumer genetic tests (DTC-GT) have become a bridge between marketing and traditional healthcare services. After earning FDA endorsement for such facilities, several fast-developing companies started to compete in the related area. Pharmacogenomic (PGx) tests have been introduced as potentially one of the main medical services of such companies. Most of the individuals will be interested in finding out about the phenotypic consequences of their genetic variants and molecular risk factors against diverse medicines they take or will take later. Direct-to-consumer pharmacogenomic tests (DTC-PT) is still in its young age, however it is expected to expand rapidly through the industry in the future. The result of PGx tests could be considered as the main road toward the implementation of personalized and precision medicine in the clinic. This narrative critical review study provides a descriptive overview on DTC-GT, then focuses on DTC-PT, and also introduces and suggests the potential approaches for improving the clinical related outcomes of such tests on healthcare systems.

## 1. Introduction

### 1.1. Direct-to-Consumer Genetic Tests 

Since the completion of the Human Genome Project (HGP), DNA sequencing tests for health-related purposes have become common in medical laboratories [1,2]. Additionally, the advent of high-throughput sequencing methods has made DNA analysis tests faster and easier. During early 2000, some genetic and genomic companies started offering genetic testing directly to individuals without the need for the prescription of physicians or other healthcare providers. Direct-to-consumer genetic tests (DTC-GT) are defined as genetic tests marketed directly to customers through print or visual media or the internet, or that can be bought online or in brick-and-mortar stores with no/least involvement of healthcare professionals in this process [3]. DTC-GT allows customers to access their genomic interpreted data whenever and wherever they want. However, over time, after some critical evaluations, companies selling DTC-GT started engaging geneticists and medical professionals’ in the form of pre-and post-test consultation for consumers (advertisements, articles, brochures, personal contact, etc.) to provide advice on further actions to be taken after the genetic testing results. Most companies declare in their policies and consent forms for consumers that such tests should not be considered as diagnostic tests, but only as informative tests [4]. Although the DTC-GT tests will be more useful in preventive areas than diagnosis issues, the test outcomes can demonstrate significant help for the future clinical assessments of individuals through providing the healthcare-related result before they visit a specialist. A DTC-GT report can bring attention to a specific condition in people, which may need more consideration and clinical confirmation or intervention by the clinicians [5]. 

Today, DTC-GT services are available as kits for obtaining saliva or buccal swab samples which are non-invasive. The samples can then be sent to the company providing the service where DNA analysis is performed, usually in CAP and/or CLIA accredited laboratories. The companies mostly using array-based or sequencing platforms (targeted gene sequencing panels or broad range genomic tests such as whole-genome sequencing (WGS) or whole-exome sequencing (WES)) approaches for analyzing specific mutations or providing a comprehensive picture for genomic variants in a given sample. Each company applies its microarray or sequencing technologies (i.e., Illumina HumanOmniExpress-24 single nucleotide polymorphism (SNP) chip for 23andMe and WES for Genos) with post-processing involving imputation, and the interpreted genomic data then are returned to customers via the internet or mail after a couple of weeks or months [6]. Some companies such as 23andMe (Sunnyvale, CA, USA), Color Genomics (Burlingame, CA, USA), etc. also provide raw genetic data to their customers, so the customers can use this raw genetic data for further processing and analysis through free online resources and tools such as Promethease, Live Wello, Genetic Genie, etc. with the help of a physician, clinical geneticist, genetic counselor, pharmacist or other trained genetic professionals. 

Currently, DTC-GT companies offer their services in two main categories which include medical and non-medical genetic tests. The medical genetic tests can be classified as carrier tests (e.g., hemoglobinopathies), disease susceptibility detection tests (e.g., Parkinson’s disease), pharmacogenomic tests (for the specific number of drug-related genes), life-style related tests (genetic analysis for complex diseases), and prenatal tests (PND & PGD). They can also be divided into tests for monogenic disorders, polygenic defects, multifactorial diseases, genome-wide testing (thousands of SNPs), and broad range tests (WGS & WES). Medical genetic testing services are the most common tests used by people and are the main reasons for the increasing growth of DTC-GT companies. Non-medical testing services, on the other hand, consist of testing for some traits and features in individuals, which are not necessarily related to disease or health, and are usually for “infotainment”. Examples of these include ancestry information, ear lobe attachment, and the flush reaction after drinking alcohol, etc. However, there is an argument that ancestry data should be included under medical information, because this information helps to determine whether a specific ethnic group has a predisposition to a particular genetic condition (e.g., Tay-Sachs disease in individuals of Ashkenazi Jewish ethnicity and lactose intolerance in people of East Asian, West African, and Arab descent) [7]. This study aims to provide an overview of direct-to-consumer pharmacogenomic tests (DTC-PTs) as one of the health-related services for DTC-GT companies and discuss the strategies that might be beneficial for improving the market usability and clinical outcomes of such tests.

### 1.2. Pharmacogenomics and Its Integration in DTC Companies

Pharmacogenomic (PGx) tests constitute one of the important genetic testing services of DTC-GT companies. PGx tests reveal genetic variations that can be linked to the efficacy and/or responses to drugs; therefore, most people are interested in finding out about their genome function concerning their drug intake. As a potential molecular risk factor, PGx variants may affect several medication processes and bring about the different outcomes of safety and efficacy for assigned treatment approaches. Studies have reported that almost all people have at least one actionable functional variant in their genes for drug pharmacokinetics and pharmacodynamics [8]. Most pharmacovariants are categorized as polymorphisms through the human genome; therefore, they may show no discernable phenotype until the time for drug utilization by individuals. Hence, pre-emptive genotyping and providing the result (by DTC companies) would be extremely beneficial for the patients who refer to the clinic later. Indeed, drug-related gene scanning can provide the information before any prescription and clinical decision. Besides, the PGx test data can be used as a lifetime predictive tool for drug safety and efficacy. PGx profiling (not as DTC) is a routine test in some clinical laboratories and hospitals (e.g., Mayo Clinic, St. Jude Children’s Research Hospital, Vanderbilt University Medical Center, etc.) and soon it will become prevalent in many clinical centers through the updating of different provided guidelines [9]. As the global need for PGx tests is increasingly acknowledged [10,11], more DTC-GT companies also will begin to provide PGx testing services in the near future. 

### 1.3. Direct-to-Consumer Pharmacogenomic Tests (DTC-PT)

Various companies are offering several different genetic tests and services, but some companies are offering only a few specific tests. Currently, PGx analysis of individuals as pre-emptive genetic profiling and screening is offered by just a few companies (Table 1). Based on companies’ public pages and depending on test type, whether it is single, combined with other tests, or a whole genome test, the price ranges from USD 100 to USD 1000 in different centers. The various functional genetic variations (FGVs) in the genome are determined so that proper prescription and treatment decisions can be provided; this helps in realizing the dream of personalized and precision medicine (PPM). 

PGx tests were launched for the first time in the early 1990s, with the anticipation that they could be used as an approach for reducing many potential adverse drug reactions (ADRs) and the first FDA approval of such tests appeared in 2005 [13]. Similarly to other DTC genetic tests, DTC-PTs also are evaluated and assessed by the FDA through monitoring the clinical and analytic validity in addition to consumer’s understanding and perception of the descriptive information for the tests and the related results without any professional healthcare intervention. The regulations for the test implementation are then managed and declared to the companies subsequently. 

As per the two pioneering companies in this field, 23andMe and Pathway Genomics, more than 91% of their customers showed FGVs [14]. Today, different companies evaluate and profile different drug–gene pairs. Even the screening portfolio for a single company may vary in different countries. For example, 23andMe, the only DTC-PT company with FDA approval for three PGx markers without a physician’s prescription, has provided profiling for different genes in different countries before [15]. This is because people with diverse ethnicities show different types of biomarkers for the same drug which could result in alternative responses and efficacy. 

Concerns have been raised by some civil society organizations and regulatory bodies such as the FDA about the lack of medical supervision for most of the DTC PGx tests, resulting in a reduction in the number of companies offering these services [16]. While the industry was reshaped and down-sized by the FDA warning letters, the need to obtain clearance/approval for such tests placed was as the top priority issue for the offering companies. At present, a few companies are offering PGx tests, either directly to consumers (23andMe) or through a physician (e.g., Veritas) [17,18,19]. However, because of the waiting time for an appointment with a healthcare provider to order the PGx tests, people are reluctant to spend time receiving test orders from the physicians. Hence, companies that offer PGx tests directly to consumers may become more popular and will become the most common mode of PGx testing in the future; especially when such tests can be organized as a pre-emptive genotyping approach for individuals. Such companies should provide additional information to both patients and physicians before the test. The interpreted data of tested PGx biomarkers and related literature alongside the test methodology should be provided by the companies on their websites so the essential scientific information will be available for customers before they order PGx tests [20]. The results of PGx profiling by DTC companies may serve as an approach for increasing the efficiency of future prescribed drugs. Even though the test is performed only once, the results can be utilized for people’s whole lifetime. Below are some insights into the field which could improve the market usability and clinical outcomes for these tests.

### 1.4. Approaches to Improve the Clinical Outcomes of DTC-PT

DTC-PT is a double-edged sword, because it can raise concerns about drug dosage adjustment if the results are misinterpreted but can truly help if handled properly. We observed the pitfalls of DTC-PT tests over time and propose different strategies to improve their clinical outcomes alongside the market usability. Here, we list some of the main challenges that should be addressed appropriately to improve the positive effects of the DTC-PT. The first and, maybe the most important of all, is the integration of physicians and other healthcare providers such as human/clinical geneticists and clinical pharmacologists in the test procedure because they can provide appropriate scientific and clinical information about the test itself and the results to consumers [21]. This will improve the completeness and reliability of such companies. However, it has been stated that the final decision about ordering the tests should still be made by the customers themselves; the current PGx guidelines are just about the interpretation of test results and not about who should order the tests, or when [22]. Companies can make information easily available to customers through advertisements, articles, brochures, personal contact, etc. However, most companies provide information about analytical and clinical validity and utility and test quality of DTC-PT through in vitro diagnostic (IVD) validated equipment (i.e., premarket approval code for approved devices if there is one) for the physicians, who ordered the tests. Such diagnostic tests also have been described earlier and are freely accessible to everyone [23]. Then, all the companies which provide PGx profiling may consider and prepare relevant information for customers alongside specialized information for healthcare providers. 

After gaining public trust by providing the needed information to consumers, the companies’ efforts could be focused on personalizing the services. PGx variants may be highly dependent on the specific population; therefore, different alleles plus population-specific haplotype/diplotype for every pharmacogene must be considered in their tests. This will also make a huge impact on the clinical validity and utility of the tests [24]. Currently, many companies use pre-prepared SNP array chips or different orthogonal PCR approaches as genotyping methods (23andMe, Genelex (Seattle, Washington, United States), etc.). Here, the different allele frequencies and linkage patterns between different ethnic groups must also be considered for PGx result analysis. For example, the frequency of poor metabolizer alleles for *CYP2C19* is higher in East Asian ethnicities (14%) when compared to those with European (2%) and African (4%) ancestries. Even in those variants which were considered to have a relationship with specific drugs universally, it has been found that there is an effect of ethnicity. Some of the examples are warfarin and rs9923231 in the *VKORC1* gene and abacavir and the HLA-B*57:01 allele [15]. In such a scenario, the recommendation is the employment of hypothesis-free sequencing technologies (WES, WGS, or long-read sequencers) besides using any local variant datasets for obtained data interpretation. This might be necessary when there are no clear guidelines from reference organizations concerning the identified variants, only annotations. However, any type of existed reference data could be provided alongside the final result for the customers, who are recommended to consult with a clinician based on their test results. To incorporate the revealed FGVs into clinical practice, some important factors and information such as sample numbers, ethnic background, and efficacy rate on dosage modification must be considered by a referred physician [25]. New approaches for companies to deal with these issues could include the integration of an interdisciplinary team consisting of different fields of study in companies’ properties; as such, team efficacy for patients’ safety has been reviewed before [26]. The related team could comprise a clinical geneticist, laboratory geneticist, clinical pharmacologist, and a medical doctor in the test-providing group (scientific support section (SSS) in the company). Gathering such professional medical advisors maybe not widely available; therefore, the feasibility and economic implications of DTC companies for implementing the recommendation from SSS members alongside the tests for the customers could also be a matter of challenge for some corporations. However, it would increase the credibility of the PGx test service if those companies utilized the services of SSS before providing results to their customers. Moreover, the interpretation and follow-up recommendations can be provided by the SSS team. It will also be useful for the companies if the SSS members can engage with the scientific research community and scrutinize the provided publications to gain new insights into PGx tests [27]. Other approaches to improve the quality of companies’ services include the preparation of a well-designed personalized electronic card containing PGx test results which can be accessed quickly and made available through a linked local FGV database [28].

Finally, there are some general trends for providing optimal and comprehensive PGx test outcomes. The first is increasing the numbers of included pharmacovariants (either with a guideline or annotated) into the test by employing next-generation DNA sequencing or long-read sequencing technologies instead of current techniques, as well as SNP arrays for variant identification. For example, 23andMe mostly utilizes its SNP chip for genotyping 715,000 SNPs [15,29] but their tests are incomplete because many new and/or previously reported informative variants for some main pharmacogenes have not been captured in their panel. For instance, the panel ignores some population-specific predictive PGx markers in *HLA-B*, *IFNL4*, and *TPMT* genes. 23andMe however, reduced the number of pharmacogenes and involved variants significantly, as is mentioned in their related PGx portal [12]. The screening and inclusion of new variants into the company’s medical and health-related tests also need a license from the relevant authorities. Furthermore, because several PGx markers can be found in intronic and regulatory elements of pharmacogenes and the presence of some insertion–deletions (InDels), copy number variations (CNVs), and pseudogenes in drug-related genes (e.g., *CYP2D6*), next-generation sequencing (NGS) and long read sequencer technologies would be the best choice for identifying such variants [30]. Comprehensive NGS methods such as WES and WGS work as hypothesis-free approaches and will find most of the potential FGVs in drug genes. The incidental findings (IFs) and variants of unknown significance (VUS) could be ignored in the final result, because the DTC companies’ goals and general policies do not enter in research or diagnostic areas but only identifying those variants which have been offered for detection before. Nevertheless, the challenging variants may be followed for further analysis by the SSS teams in companies. The second approach for optimizing and improving DTC-PT services is having a list of most prescribed drugs locally and focusing on the related pharmacogenes. It will make the tests outline the personalized and precision medicine (PPM) area more than before. Probably, in this way, the result shows more annotated variants than those with a clear guideline. In this case, companies should add a disclaimer to their reports that there might be a limitation and changeable efficacy for a particular result. The third and last approach would be the participation of the company’s representatives in scientific events to obtain scientific credentials in the field. At present, most companies also seek customers’ informed consent to share the customers’ data. Such activities will fuel research and bring more credit and validity to companies [31]. For instance, according to 23andMe, more than 80% of their customers agreed to use their genomic data in the medical research area. However, strict informed consent forms and clear and sufficient information on further activities by the companies must be provided before this [32]. The approaches for improving the clinical outcomes of DTC-PTs are summarized in Table 2. 

### 1.5. Ethical and Legal Considerations for DTC-PT

DTC-PT by definition includes no healthcare supervision in the test procedure. In the last decade, DTC companies have made it easier for people around the world to access DTC PGx testing; however, the lack of clinical supervision has raised many concerns, especially when changes in drug ordering, dosage adjustment, and other treatment approaches are required, in addition to customers performing self-therapy. These concerns have made DTC-GT, and of course the PGx tests through the related companies, controversial since their advent in the market [4]. Over time, many regulatory and legislative entities such as the FDA, EU parliament, and U.K. Human Genetics Commission have started to monitor and implement regulations and directives for such tests [33]. In 2013, the FDA warned 23andMe to wait for pre-marketing assessments and approval laws for their tests. Based on the warning letter, the company ceased and desisted the PGx tests that were offered. However, in 2017, the FDA sent an approval letter to the company for including some specific personal genomics and health-related tests (involving PGx profiling). Currently, 23andMe is the only company which offers PGx tests that can be requested directly by the customers. However, the rules are different between countries. For example, in European countries, laws were enacted both at the national and EU levels. These regulations are described in detail in other publications [32,34,35]. However, questions such as why the number of FDA-revised and approved pharmacogenetic biomarkers differs from those which could be offered by the DTC companies, remain unanswered regarding DTC-PT implementation through the offering centers. 

Additionally, consumers’ data storage and future utilization in other activities or shared with third parties would be a very important concern in customers’ privacy protection and confidentiality. While the recommendations and guidelines for DTC-GT health-related services are available in the statements of policymakers and observers as well as the European Society of Human Genetics (ESHG), Global Alliance for Genomics and Health (GA4GH), Nuffield Council on Bioethics (NCB), etc., the information on data storage times, sample disposal, and extra research activities still varies between different companies; unfortunately, some do not consistently meet the international guidelines on transparency, related to privacy and secondary use of customers’ data [36]. Hence, the consumers’ privacy protections and expectations must be handled with care by the companies’ terms of use, laws, and regulations. Recommendations for this public controversy have previously been provided and highlighted, which may raise advanced discussions in the field [37]. Table 3 also lists some important considerations for companies. 

## 2. Discussion and Future Trends 

Today, PGx studies provide a lot of information from drug–gene pairs. Just a couple of years ago, fewer than 80 drugs had PGx tags, but now there are more than 260 unique drugs with PGx recommendations, available through organizations such as CPIC, DPWG, CPND, and the FDA [38,39]. The labels include actionable PGx, testing required, testing recommended, and informative PGx. The latter, according to PharmGKB, means that particular variants or metabolizer phenotypes do not affect a drug’s efficacy, dosage, metabolism, or toxicity. However, the lack of sufficient education for physicians and inaccessibility to such tests alongside lack of insurance coverage are major issues for customers and are barriers to making PGx profiling a routine and mandatory test. Additionally, inconsistencies in clinical pharmacogenetic recommendations among major sources exist, which may slow the clinical implementation of such test results. The prevalence and type of these inconsistencies have been comprehensively analyzed before [40].

Over time, factors such as pharmacogenetic information accessibility before physician order and the pre-symptom identification of PGx biomarkers helping to provide better personalized clinical decision-making thereby reducing drugs’ side effects and adverse reactions, etc., has led to the rise of DTC PGx testing [3,10]. However, challenges such as clinicians’ lack of education to understand the results, the non-availability of a geneticist or genetic counselor in most clinics, the interpretation of results based on just the previous genome-wide association studies, no consideration of family and background risks and people’s lifestyle, ethical and legal issues, the utility and validity of tests, potential misinterpretations of results, possible wrong decisions by healthcare providers due to the incorrect interpretation of results, and a lack of adequate clinical evidence for most identified FGVs led to the downfall of DTC-PTs [18,41]. However, after the FDA approval letter to a company including some PGx tests among other tests, PGx tests came back into the limelight and PGx genotyping started to rise again. Nowadays, there are some companies that perform DTC-PTs for their customers as one of the main options between other healthcare-related tests (Table 1), although, there are still some scientific considerations that must be addressed by these companies. For example, many genetic effects and modifications such as the presence of rare recombination events in specific populations, dominant negative effects of mutations in genes in drug metabolic pathways, gene duplication occasions in populations, epigenetic signatures in people, epistasis occurrence, variable expressivity through intra- and inter-families, and incomplete penetrance effects for some genetic alterations receive no attention during PGx tests. Therefore, the provided results may not depict the true potential of pharmacogenetic profiling and functioning of individuals [42]. Additionally, different specificity and sensitivity, mainly because of diverse genetic variations or allele/haplotype frequencies for the tested pharmacogenes, in addition to the presence of any linkage disequilibrium between the tested SNPs in population-specific panels and possible findings through comprehensive genotyping approaches such as WES and WGS, plus long-read sequencer outcomes, should be taken into account. If any companies would like to be the pioneer and/or frontier in providing the most accurate DTC-PT services among the others, such a genetic analysis must be handled via the SSS team before declaring the final result to customers or their healthcare providers [43,44]. For FGVs with the guideline, the task is clear for incorporation into clinical practice, but for the annotated variants more research evidence is required. Here, companies may use different approaches for reaching this goal. For instance, 23andMe considered at least three papers for considering the variance in their PGx testing [45]. The challenge is when the clinical relevance of the variant is proved in just one published document. However, during the provision of personalized treatment for patients, it is better to also consider a clinicians’ opinion, which might be the exact genetic alteration for ADR in the specific patient(s). Nevertheless, alongside all these issues, companies must always remember that they should not cause any unnecessary concerns or anxiety for people.

There were no legal concerns about the DTC PGx tests before, which meant that many people were willing to perform genetic profiling for themselves. This indicates the consumers’ desire for such tests and is a reminder of the fact that if there were proper regulations and directives by the governmental and legislative bodies, both patients and doctors would use PGx data for personalized prescribing, especially for the high-risk gene–drug pairs. Nevertheless, some basic issues as well as the lack of evidence-based guidelines for the use of PGx testing, such as the potential liability if prescribers do not consult PGx test results for every medication prescribed, if non-affordable medication is indicated by the customers’ PGx test results, or if there is no access to the medication recommended. Today, there is an increasing trend for using bioinformatic tools and frameworks without any need for background knowledge of complicated programming languages and scriptwriting for Linux/Ubuntu systems or Python (SUSHI of ETH Zurich, VarSeq of Golden Helix, etc.) [46,47]. Therefore, the utilization of high throughput sequencing technologies and data analysis and interpretation has become easier and more common for companies. Because of that, more DTC companies may use comprehensive genotyping approaches. Soon, PGx tests may be ordered by customers and final results will be available before visiting their doctors, making treatment decisions faster. 

## 3. Conclusions 

The authorities’ regulations and the companies’ trends for providing different DTC genetic services are changing rapidly. DTC-PT may show the potential impact for becoming an essential tool for providing drug-related genetic variation information. More support in the form of funding (NIH), education (Gene-Equip, NICE), and dataset preparation (Illumina, San Diego, California, United States) are expected soon [48]. Advances in technology bring a broader range of gene–drug interactions to companies’ local panels. Web-based interpretation services and smartphone applications, as well as cost lowering for PGx tests, make them very common and accessible everywhere. Lifetime and free consultations of DTC-PT results will be offered by these companies. In this exciting area, improving the clinical related outcomes and market usability of PGx tests could be guaranteed by the SSS members of companies. Soon, we may witness the smart future of PPM, where the pre-emptive PGx tests apply as routine tests by a majority of the population. 

## Figures and Tables

**Table 1:** List of direct-to-consumer genetic tests DTC-GT companies offering pharmacogenomic PGx tests.

Company	Status of the PGx Test	Covered Pharmacogenes	Covered Pharmacovariants
23andMe	Directly by the customers	*CYP2C19*	*2, *3, *17
		*DPYD*	*2A, rs67376798
		*SLCO1B1*	*5, *15, *17
Veritas Genetics	Ordered by the physicians	Not available	Not available
Genelex	Ordered by the physicians	*CYP2C9*	*2, *3, *4, *5, *6, *8, *11, *13, *15
		*CYP2C19*	*2, *3, *4, *5, *6, *7, *8, *9, *10, *12, *17
		*CYP2D6*	*2, *2A, *3, *12, *14, *15, *17, *19, *20, *21, *29, *30, *35, *36, *41, *56, *109
		*CYP3A4/CYP3A5*	4*22, 4*1B, 5*3, 5*6, 5*7
		*ADRA2A*	rs1800544
		*CYP1A2*	*1A, *1C, *1E, *1F, *1J, *1K
		*CYP2B6*	*2, *4, *5, *6, *7, *9, *16, *18, *28
		*CYP4F2/VKORC1*	c.1297G>A/c.-1639G>A
		*COMT*	c.472G>A
		*DPYD*	*2, *13, c.2846A>T
		*Factor II—Factor V Leiden*	Factor II: c.*97G>A (g.20210G>A) Factor V: c.1601G>A (c.1691G>A)
		*GRIK4*	c.83-10039T>C
		*HLA-A or HLA-B*	A*31:01, B*15:02, B*57:01 rs2395029, B*58:01
		*HTR2A*	c.-998G>A (c.-1438G>A), c.614-2211T>C (c.1178G>A)
		*HTR2C*	c.-759C>T
		*IFNL3*	rs12979860C>T
		*MTHFR*	c.665C>T (c.677C>T), c.1286A>C (c.1298A>C)
		*NAT2*	*4, *5A-E, *5G, *5J, *6A-C, *6E, *7A, *7B, *11, *12A-D, *13, *14A-G, *19
		*OPRM1*	c.118A>G
		*SLCO1B1*	*1B, *5, *9, *14, *15, *17, *31, *35
		*TPMT*	*2, *3A–C, *4
		*UGT1A1*	*28
Pathway Genomics	Ordered by the physicians	*GRK5*	rs17098707
		*ADRB1*	rs1801253
		*CYP1A2*	rs762551
		*CYP2C19*	rs4244285, rs4986893, rs12248560, rs28399504, rs41291556, rs56337013, rs72552267, rs72558186
		*F2*	Prothrombin G20210A
		*F5*	Factor V Leiden
		*CYP2D6*	rs16947, rs769258, rs1065852, rs1080985, rs3892097, rs5030655, rs5030656, rs5030865, rs28371706, rs28371725, rs35742686, rs59421388, rs72549357, rs5030862, rs5030863, rs5030867, rs59421388, rs35742686
		*AGTR1*	rs5182, rs275651
		*BDKRB1*	rs12050217
		*SLCO1B1*	rs4149056
		*NOS1AP*	rs10494366
		*CACNA1C*	rs1051375
		*CYP2C9*	rs1057910, rs1799853, rs9332131
		*VKORC1*	rs9923231
		*CYP2B6*	rs2279343, rs3211371, rs3745274, rs8192709, rs28399499
		*CYP3A4*	rs4646438, rs35599367, rs55901263, rs55951658, rs67666821, rs138105638
		*CYP3A5*	rs776746
		*DRD2*	rs1799732
		*HLA-A*	rs1061235
		HLA-B *1502	rs2844682, rs3909184
		*HTR2A*	rs7997012, rs6311
		*HTR2C*	rs1414334, rs3813929
		*POLG*	rs113994095, rs113994097, rs113994098
		*SLC6A4*	5-HTTLPR, rs25531
		*UGT1A4*	rs2011425
Genos	Ordered by the physicians	Whole Exome Sequencing but not interpreted	Containing all drug related genes
Theranostics	Ordered by the physicians	*CYP2C19*	*2, rs4244285, *3, rs4986893, *17, rs12248560
		*ABCB1*	rs1045642
		*CYP2C9* *VKORC1*	*2, rs1799853, *3, rs1057910 rs7294, rs9923231
		*CES*	rs4148738
		*SLCO1B1* & *ABCG2*	rs4149056, rs2231142, rs2306283
		*SORT1*	rs599839
		*PCSK9*	rs11206510
		*MIA3*	rs17465637
		*PHACTR1*	rs12526453
		*LPA*	rs3798220, rs10455872
		*ABO*	rs579459
		*CXCL12*	rs501120
		*APOA5*	rs964184
		*HNF1A*	rs2259816
		*SH2B3*	rs3184504
		*LDLR*	rs1122608
		*IL6R*	rs4845625
		*GGCX/VAMP8*	rs1561198
		*ABCG8*	rs6544713
		*APOB*	rs515135
		*SLC22A4/SLC22A5*	rs273909
		*SLC22A3/LPAL2/LPA*	rs2048327
		*PLG*	rs4252120
		*CDKN2BAS*	rs3217992
		*CXCL12*	rs2047009
		*FLT1*	rs9319428
Color	Ordered by the physicians	*CYP1A2*	*1F, *1J, *1K
		*CYP2D6*	*2, *3, *4, *4N, *5, *6, *7, *8, *9, *10, *11, *12, *14A, *14B, *15, *17, *19, *29, *35, *36, *41, *xN
		*CYP2C19*	*2, *3, *4A, *4B, *10, *17
		*CYP2C9*	*2, *3, *4, *5, *6, *8, *11
		*CYP3A4*	*1B, *22
		*CYP3A5*	*3, *6, *7
		*CYP4F2*	*3
		*DPYD*	*2A, *13
		*F5*	rs6025 (Leiden)
		*IFNL3*	rs12979860
		*NUDT15*	rs116855232
		*SLCO1B1*	rs4149056
		*TPMT*	*2, *3A, *3C, *4
		*VKORC1*	rs9923231

The information provided in this table is either adapted from the companies’ official websites, or by direct contact to the authorities of such companies. 23andMe data were obtained through the 23andMe Pharmacogenetics portal [12]. * A standardized nomenclature system used for various haplotypes and alleles in pharmacogenes.

**Table 2:** Challenges and suggestive strategies to improve the clinical outcomes and market usability of DTC-PT.

Challenges	Approaches
Lack of integration of healthcare professionals in the test procedure	Incorporation of a scientific interdisciplinary team as company members (see the text for details)
Self-management of the results by customers themselves or any unaware healthcare provider	Pre- and post-test counseling by the SSS* team for the customers
Lack of information in guidelines about who should go for the tests, and when	Providing this information in a manner easily accessible to customers
Absence of any information on DTC-PT* analytical and clinical validity and utility plus test quality for the ordering physicians	Providing the related information by the companies via short videos, brochures, etc.
Lack of the local and newly identified genomic variants in test panels (mostly including a limited set of known variants)	Permanent including population-specific and newly introduced variants to each test through the utilization of more advanced sequencing approaches (NGS*) instead of limited methods such as single nucleotide polymorphism (SNP) array for genotyping
Updating the knowledge of PGx* of scientific members of companies	Engagement of SSS members in scientific research projects through connection with academic members.
Fast accessibility and extensive availability of the customer’s drug–gene information everywhere	Preparing a safe and data protected well-designed personalized electronic card, linked to the local FGV database
Increasing the numbers of included variants (with guideline and/or annotated) in the PGx test	Utilization of comprehensive NGS platforms such as whole-exome sequencing (WES) and whole-genome sequencing (WGS) and long-read sequencing technologies for identification and including different type of actionable or informative variants Be notified for updated publications in the field
Providing more personalized services for customers	Preparing the list of most prescribed drugs locally and focusing on the related pharmacogenes
Obtaining more scientific credentials in DTC-PT	Participation of company representatives in authentic scientific communities and events (see the text for details)
Ethical and legal considerations for adding new PGx tests and services	Including a medical lawyer as the company’s member
The motivation for social education on PGx	Increasing public advertisement for PGx tests by companies

SSS*, scientific support section; DTC-PT*, direct-to-consumer pharmacogenetic test; NGS*, next generation sequencing; PGx*, pharmacogenomics.

**Table 3:** Ethical and legal considerations for companies offering DTC-PT.

Important Considerations
Adequacy of pre- and post-counseling
Scientific and applied validity of the tests
Prevention of misleading advertisements
Informed consent for the reuse of private PGx data (research and commercialization)
DTC-PT in minors
Deal with reporting variants with no clinical utility
Necessity of follow-up for special results
Consistency and harmonization in internal marketing
Increasing the consumers’ support
Data protection and product safety

## Data Availability

Not applicable.

## Abbreviations

ADR	Adverse Drug Reaction
CAP	College of American Pathologists
CLIA	Clinical Laboratory Improvement Amendments
CPIC	Clinical Pharmacogenetics Implementation Consortium
CPND	Canadian Pharmacogenomics Network for Drug Safety
DPWG	Dutch Pharmacogenetics Working Group
DTC-GT	Direct-to-Consumer Genetic Test
DTC-PT	Direct-to-Consumer Pharmacogenomic Test
ESHG	European Society of Human Genetics
FGVs	Functional Genetic Variations
GA4GH	Global Alliance for Genomics and Health
HGP	Human Genome Project
IF	Incidental Finding
IVD	In Vitro Diagnostic
NGS	Next-Generation Sequencing
NCB	Nuffield Council on Bioethics
PGx	Pharmacogenomics
PPM	Personalized and Precision Medicine
SNP	Single Nucleotide Polymorphism
SSS	Scientific Support Section
VUS	Variants of Unknown Significance
WES	Whole Exome Sequencing
WGS	Whole Genome Sequencing
